# Investigation of Spent Moulding Sand Using Thermal Treatment with Regard to the Possibility of Recovering Quartz Matrix

**DOI:** 10.3390/ma17235991

**Published:** 2024-12-06

**Authors:** Mariusz Łucarz, Aldona Garbacz-Klempka, Marcin Brzeziński, Alena Pribulová, Patrik Fedorko

**Affiliations:** 1 Faculty of Foundry Engineering, AGH University of Krakow, Reymonta 23 St., 30-059 Krakow, Poland; agarbacz@agh.edu.pl (A.G.-K.); mbr@agh.edu.pl (M.B.); 2 Faculty of Materials, Metallurgy and Recycling, Technical University of Kosice, Letna 1/9, 042 00 Kosice, Slovakia; alena.pribulova@tuke.sk (A.P.); patrik.fedorko@tuke.sk (P.F.)

**Keywords:** foundry waste, spent sand, scanning electron microscopy, thermal analysis, analysis of the grain surface

## Abstract

The ongoing sustainable reduction in natural resources is prompting companies to look for materials to reuse that were previously classified as waste. Uses are sought for them either in their existing area of use or in other areas of the economy. In many cases, this is difficult. The aim of this research is to see if there is a possibility of reusing the grain matrix, a major component of spent moulding sand that was perhaps diverted too early as waste to landfill. This study included three samples of spent moulding sand of unknown origin from landfills. A study of the collected materials was carried out to identify and characterise the impurities accumulated on the surface of the matrix grains. Sieve analyses, scanning photographs, and chemical analysis with a scanning microscope were performed. The surface morphology of the samples was assessed using a confocal microscope, and chemical composition analyses were performed using LIBS laser-induced emission spectroscopy LIBS. The thermogravimetric analysis, ignition loss, and gas formability of the tested materials were performed. The tested samples were subjected to high temperatures as the most efficient method of organic waste disposal. The analyses carried out earlier were repeated on the resulting material. It was found that only one of the tested samples, in the case of the application of thermal reclamation of spent moulding sand, allowed for obtaining a grain matrix (quartz sand) of high purity scale for reuse in the foundry industry or after further treatments in other industries. The other wastes analysed require intensive mechanical treatment, which does not guarantee, due to the shape of the grain matrix, the expected purity of the quartz sand or, in the case of intensive mechanical abrasive influences, a satisfactory grain matrix yield.

## 1. Introduction

In recent years, there have been significant advances in recycling technology that have enabled materials previously considered nonrecyclable to be processed. Examples include multilayer packaging, such as Tetra Pak, which used to be difficult to separate into individual raw materials but is now effectively recycled [1,2]. Electronic waste, previously considered difficult to recycle due to its rare metal content, is also now recycled through advanced raw material recovery processes [3,4]. Synthetic fibres, such as polyester, which were previously not commonly recycled, are now recycled by chemical methods [5,6]. In addition, car tyres [7,8], carbon fibre composites [9,10,11], and plastics such as polypropylene and PVC [12,13,14,15] are now being recycled on a scale that was impossible a dozen years ago. The recovery of precious metals, such as platinum, palladium, and rhodium [16,17,18,19,20,21], from automotive catalytic converters has also developed greatly. In general, the development of technology has contributed in many industries to reducing the amount of waste created and processing existing waste. All of this is due, on one hand, to the depletion of resources and, on the other hand, to the difficulty of obtaining them. The current trend and the requirement to act in terms of sustainability and the implementation of closed-loop economy activities is also not insignificant.

The foundry industry is also a source of a significant amount of waste in the form of spent moulding sand, of which quartz sand is a major component. Due to the price and availability of quartz sand, the spent mould sand is often landfilled after the technological process. The large amount of waste created is also related to the change in technology to more environmentally friendly ones, e.g., furan moulding sand is replaced by alkali-phenolic moulding sand [22]. Some of the spent moulding sand is transferred to other areas of the economy [23,24,25,26,27,28]. Due to the depletion of good foundry sands with the right shape and crystalline structure, ecological considerations in connection with the depletion of good moulding sands of an appropriate shape and crystalline structure, and ecological reasons, i.e., the reduction in consumption of fresh moulding sands, very important in the face of depletion of deposits of these raw materials; the reduction in the devastation of the area by sand mines; the reduction in the area of landfills occupied by spent moulding sand dumps, the reduction in the penetration of dust and toxic substances from dumps to the environment, and the protection of the landscape values of the environment [29], actions are being taken to recover them already at the production stage, as well as to obtain them from landfills.

The foundry industry uses various binding materials (inorganic) or binders (organic) to create casting moulds. A major challenge is the management of the large amount of waste produced after the casting is removed from the mould. The process of regenerating spent moulding sand to recover the grain matrix (usually quartz sand) can be implemented in different ways, depending on the binder used and the expected degree of purification. Two stages are used in spent moulding sand processing technology: the so-called primary reclamation and the secondary reclamation stages. Primary reclamation, in the first stage, mainly consists of crushing the sand to a specific fraction, sieving, and dusting [30]. In some casting processes, this is sufficient. Nevertheless, it does not guarantee reproducible production conditions, as the degree of purification of the matrix grains can vary depending on, for example, the size of the casting being made and the firing or deactivation of the binder used. More important is the second stage, the so-called secondary reclamation. In this process, the aim is to remove the spent binder as much as possible from a single grain [30]. This is not an easy process for several reasons: the grains are of different sizes, the grain surface is uneven, and impurities tend to accumulate in the depressions of the surface, making cleaning difficult. It is, of course, possible, but at the cost of yield, the loss of quartz sand due to the frictional processes used, and high energy expenditure [31].

Secondary regeneration can be achieved by two methods: wet and dry [31,32]. The wet method can only be used for materials dispersed in water, and the method of recovering the grain matrix is mainly based on Stokes’ law (different speed of particle fall). This method allows the purest grain matrix to be obtained. Unfortunately, according to analyses, this method is very expensive (installation costs—area needed to create the appropriate infrastructure: mixers, centrifuges, clarifiers, filters; energy costs—drying and then cooling of the grain matrix to the appropriate temperature) and is currently not used [30]. Works [33,34,35] present methods for recovering the grain matrix using the wet method.

In its simplest division, the dry method boils down to two methods: mechanical regeneration and thermal regeneration. The former is most often applied to inorganic binders and involves the abrasion, rubbing, or crushing of spent binder material from the surface of the grains, while the latter is applied to organic binders, which can be burnt off as a result of the binder degradation and destruction processes that occur. Solutions and implementation of mechanical regeneration are presented in articles [36,37,38,39,40,41,42,43,44,45,46,47,48,49,50,51,52,53,54,55,56,57,58,59]. The authors in papers [60,61,62,63,64,65,66,67,68,69,70] discuss issues related to thermal regeneration. In addition to the ecological aspect of regeneration of spent moulding sand, economic issues are also important. The topic of regeneration costs has been addressed in publications [29,44,51,71]. The authors in the papers note that it seems inappropriate to compare mechanical and thermal regeneration methods only for reasons of energy consumption in the broader sense. In many cases, the indication of mechanical regeneration for use seems to be unjustified, both from the point of view of sustainability and from the point of view of technological factors.

The main objective of this study of the materials taken from the landfill sites was to see if the grain matrix (quartz sand) could be brought back into circulation after appropriate measures had been taken. It could be effectively managed in a foundry or it could be used in other industries after appropriate treatment. The preliminary verification carried out was also intended to provide an answer as to what resources and expenses would have to be incurred to recover the grain matrix for various applications. As part of the investigation carried out, the impurities present on the surface of the grains were verified. Various tests were carried out on samples of the materials, and thermal treatment was treated as the primary activity for classifying the various spent moulding sand samples.

## 2. Materials and Methods

Samples of spent moulding sand of unknown origin taken from landfills provided for the disposal of spent moulding sand were used as test material. There are separate areas protected against the leaching of leaching compounds into groundwater. Samples were designated with the symbols S1, S2, and S3. As described in publications [30,71,72], specific tests are recommended to identify spent moulding sand with a given bonding material. In all cases, sieve analysis, ignition losses, and microscopic examination of surface morphology are used. More recently, due to the availability of scanning electron microscopes, semiquantitative analysis has been used to analyse the surface of matrix grains, often for a comparative assessment of the material before and after a specific treatment. For environmental reasons, gas-forming studies of individual materials are also carried out; hence, the tests were performed on the materials tested. Thermogravimetric analysis also makes it possible to characterise the type of material tested.

### 2.1. Sieve Analysis

The first parameter investigated was the sieve analysis of individual spent sands. Analysis was carried out in accordance with PN-83/H-11077 [73] on a set of sieves with mesh clearance in mm: 1.6, 0.8, 0.63, 0.40, 0.32, 0.20, 0.16, 0.10, 0.071, 0.056 and bottom. The measurement consisted of sifting two 50 g samples successively through a set of sieves. A laboratory shaker LPzE-2e from Multiserw-Morek (Marcyporęba, Poland) was used for this purpose. Each material sample was sieved for 900 s.

### 2.2. Surface Morphology

Surface morphology studies were performed using the Keyence VHX-7000N high-resolution digital microscope (Keyence Ltd., HQ & Laboratories, Osaka, Japan). The instrument model is equipped with a 4K CMOS sensor (Oxford Instruments, Abingdon, UK) and the latest optical system for depth of field and high resolution. The wide dynamic range of the imaging function enables a diverse colour palette by collecting successive images for different exposure times. Image capture carried out in this way is characterised by high precision and contrast.

### 2.3. Scanning Electron Microscopy

The assessment of the quality of the grain matrix was carried out by analysing the image of the grain shape and the presence of residual impurities on the grain surface. Scanning electron microscopy (SEM) was used for this purpose. A Tescan Mira high-resolution microscope (Brno, Czech Republic) with an FEG electron source was used for this study. The surface shape of the matrix grains was studied using solid-state detectors. Measurements were carried out in a vacuum chamber using an electron beam with an energy of 20 keV, a beam current of 15 nA, and a chamber pressure of 30 Pa, with a measurement time of 1268.7 s. EDS studies were performed in semiquantitative, surface, and patternless modes. The low-vacuum mode, dedicated to nonconductive preparations, was used for imaging. The grain topography was observed in backscattered electron contrast (BSE and BSE COMPO). This allowed the image of the material under study to be differentiated, while also making it easier to distinguish between grains and impurities on its surface (according to the atomic number Z of the elements). The images of the tested materials were taken at various magnifications. The presence of individual chemical elements was analysed in selected areas by energy dispersive X-ray spectroscopy (EDS) using an Ultim Max EDS detector from Oxford Instruments (Abingdon, UK). In addition, a map of the distribution of elements on the grain surface was made. Representative areas were selected for study using 1000× magnification.

### 2.4. Thermogravimetric Analysis

A thermal analysis test was performed to identify the material that covered the surface of the matrix grains. The test was carried out using a TA Instruments SDT Q600 thermal analyser (DSC/TGA) (New Castle, DE, USA). In the furnace chamber, the temperature was changed at a rate of 10 °C/min until 1000 °C was reached, ensuring that the organic material was completely degraded and destroyed. In each case, a sample of approximately 2 g was subjected to thermal TG analysis. The test materials were placed in alumina crucibles with a thermal resistance of up to 1500 °C.

### 2.5. Ignition Losses

The primary test for evaluating the grain matrix is ignition loss. Samples in bulk form were subjected to roasting in a SNOL 8.2/1100 resistance furnace (Narkūnai, Lithuania). The determination of the ignition loss was carried out on two samples weighed into quartz crucibles. The average of the two measurements is the result presented. The test material was heated with the furnace to an annealing temperature of 1000 °C, annealed for 2 h, and then cooled.

### 2.6. Gas Formation

Measurements of the gas-forming properties of the material samples were carried out on a test stand equipped with a tube furnace-type PRC 30M/1300 from CZYLOK (Jastrzębie—Zdrój, Poland), a peristaltic pump type BT100-2J from LongerPump (Baoding, China), and a control and recording unit. The measurement consisted of heating a tube furnace (quartz tube) to a temperature of 1000 °C, into which a ceramic boat with a sample of the material under investigation weighing approximately 2 g (weighed with an accuracy of ±0.001 g) was then introduced (outside the heating zone). Once the material sample to be analysed was introduced, the tube was sealed on one side and connected to a peristaltic pump on the other side in order to create negative pressure in the reactor and pump out the resulting gases. The sample was then introduced into the heating zone, where it was heated very quickly to the measurement temperature, and the amount of gas formed was recorded. The gas-forming results are presented as averages of the three determinations. The results of the measurements were converted to one gramme of sample mass.

**Figure 1:**
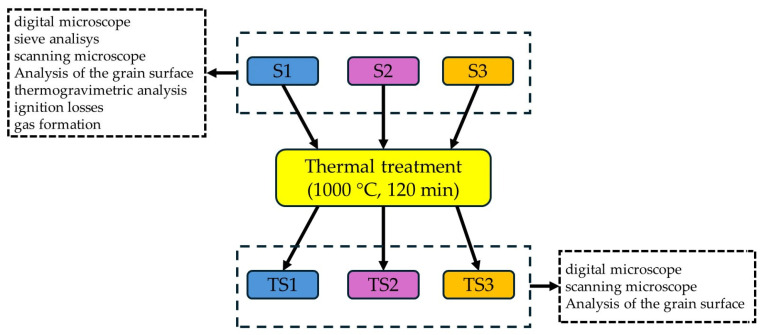
The flowchart of the research programme.

The programme of completed studies is shown in Figure 1.

## 3. Results

**Figure 2:**
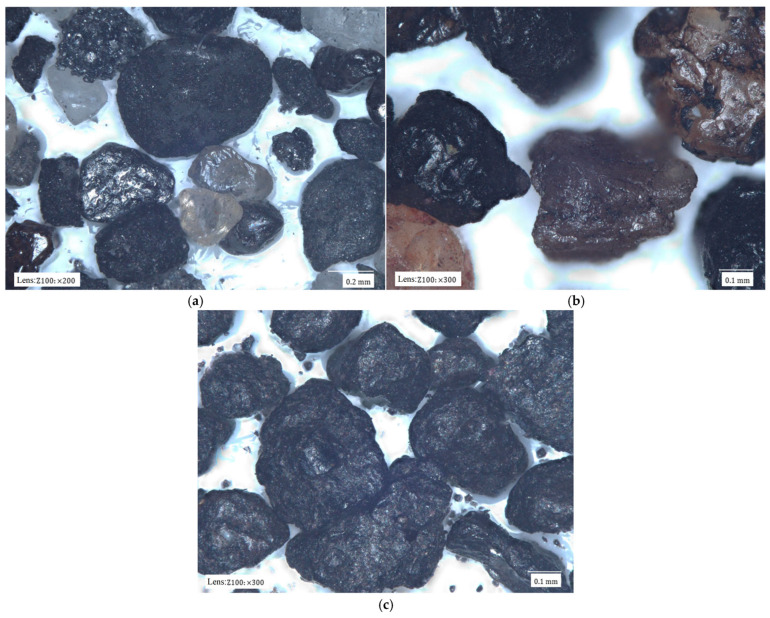
Materials analysed: (**a**) spent moulding sand S1; (**b**) spent moulding sand S2; (**c**) spent moulding sand S3.

Figure 2 shows images of the tested matrixes taken with a digital microscope.

The dark colour of most matrixes is indicative of the high contents of organic compounds that have been thermally degraded by contact with the liquid metal and the high temperature of the casting alloy. Material samples also differ in terms of grain size, homogeneity, shape, and degree of coating with spent binder. The matrix grains in Figure 2a have the surfaces of the spent binders covered to varying degrees. At the same time, they are of different sizes. It is also possible to see light-coloured grains, which may be indicative of binder burnout in contact with the liquid casting alloy. Figure 2b shows grains of similar size with very extended surfaces. These grains are also characterised by the varying surface coverage of the binder. There are grains that are completely covered with binder and those on which a significant part of the binder is in the recesses of the surface. The largest amount of spent binder can be seen in sample S3 (Figure 2c). The grains are entirely covered with spent binder. At the same time, the grains vary in size from large in the observed area to very small.

### 3.1. Analysis of the Initial Material

**Figure 3:**
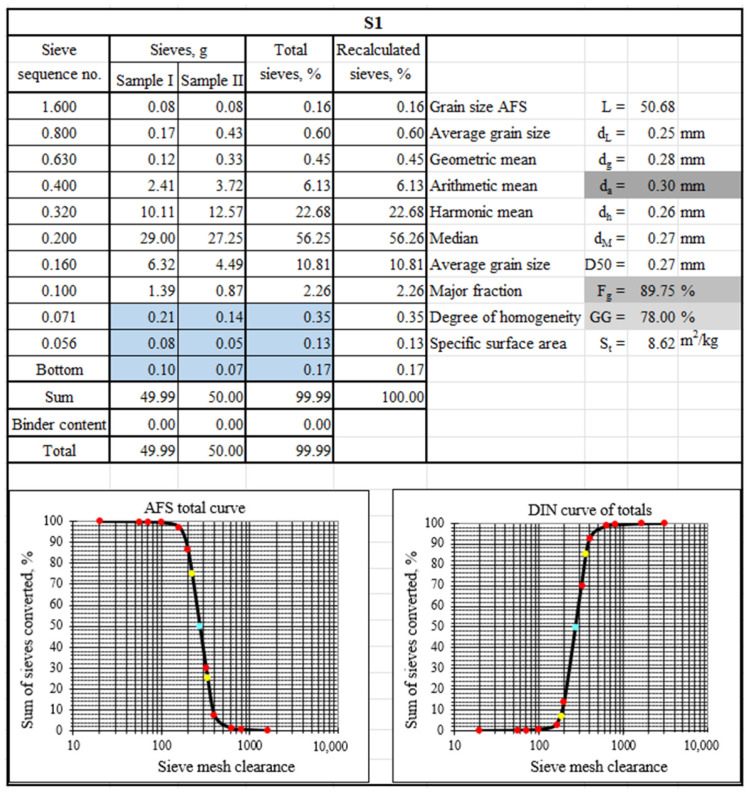
A sieve analysis of spent moulding sand S1 (blue boxes show technologically unsuitable fractions, grey boxes show sieve analysis parameters selected for comparison, coloured dots show curve determination markers).

**Figure 4:**
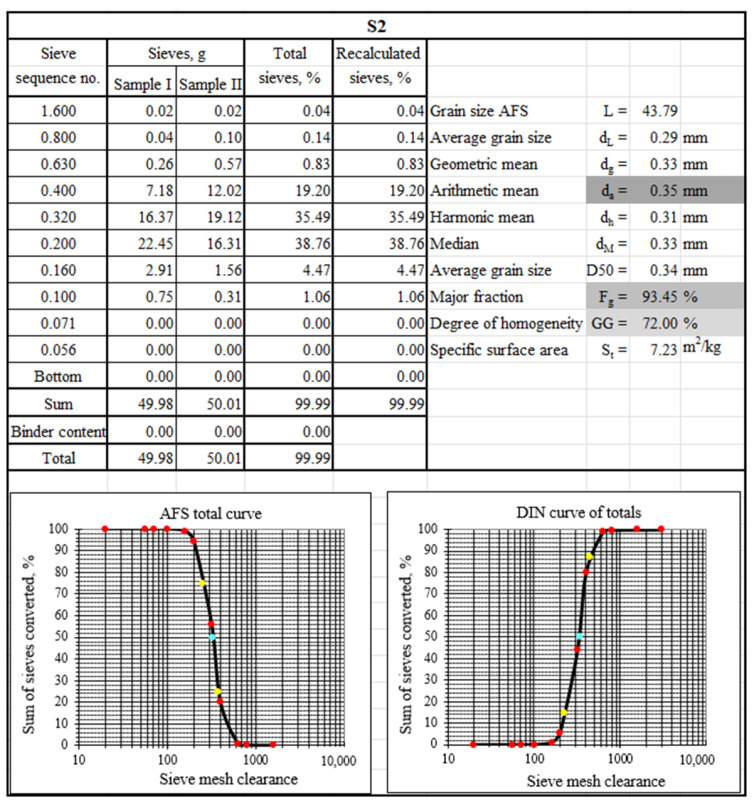
A sieve analysis of spent moulding sand S2 (blue boxes show technologically unsuitable fractions, grey boxes show sieve analysis parameters selected for comparison, coloured dots show curve determination markers).

**Figure 5:**
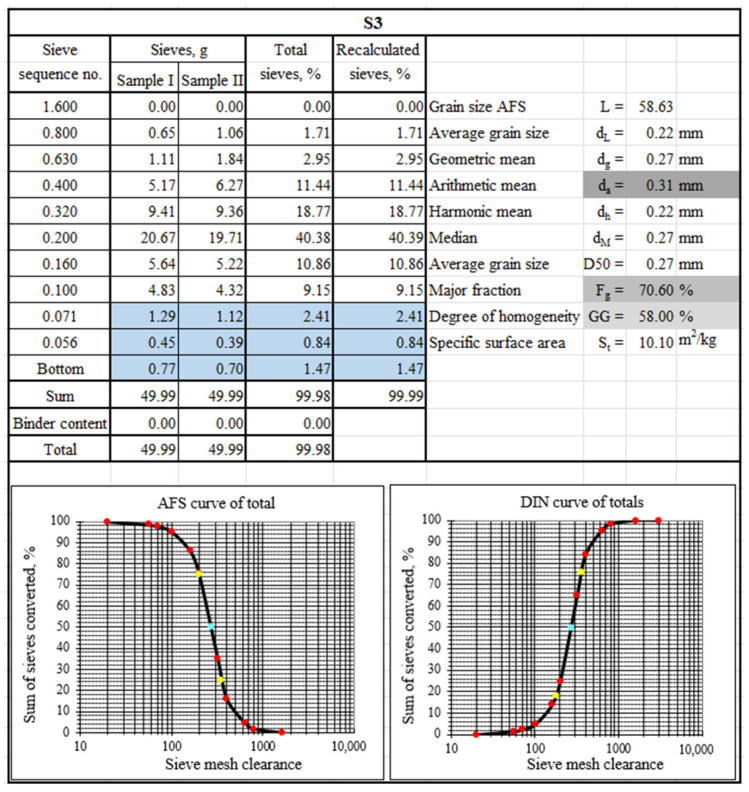
A sieve analysis of spent moulding sand S3 (blue boxes show technologically unsuitable fractions, grey boxes show sieve analysis parameters selected for comparison, coloured dots show curve determination markers).

To characterise the tested materials in terms of grain matrix size, sieve analysis was performed. Figure 3, Figure 4 and Figure 5 show the sieve analysis sheets of the tested material samples.

Figure 3, Figure 4 and Figure 5 show the selected sieve analysis parameters in grey boxes. The largest grains were characterised by the grain matrix (d_a_ = 0.35 mm), followed by S3 (d_a_ = 0.31 mm), and the smallest was the grain matrix (d_a_ = 0.30 mm). The grain matrix was the most homogeneous (GG = 78%), while the material labelled S3 had the worst performance (GG = 58%). The main fraction of sample S1 was collected in sieves (0.20/0.32/0.16), sample S2 on sieves (0.20/0.32/0.40), and sample S3 on sieves (0.20/0.32/0.40). Figure 3 and Figure 5 show with a blue background the occurrence of ‘technologically unsuitable fractions’, which are usually removed in the production process, for example, by pneumatic grading. These smallest particles impaired the permeability of the moulding sand and, at the same time, absorbed the binding material to the greatest extent.

**Figure 6:**
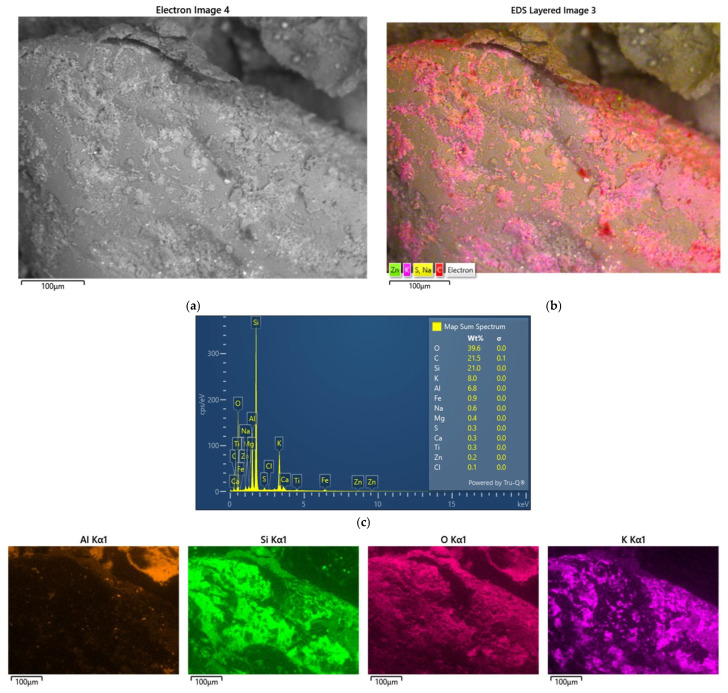

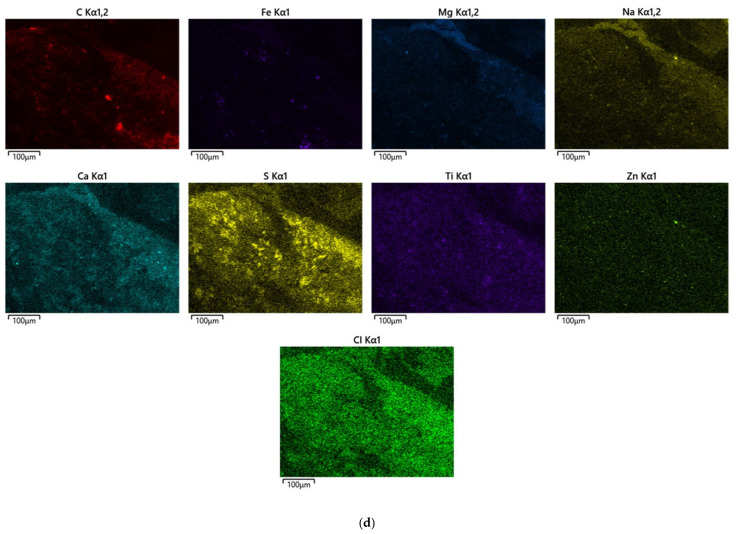
An analysis of the chemical composition of the grain surface of sample S1: (**a**) surface scanning photo, mag. ×1000; (**b**) scanning photo with elemental intensity; (**c**) spectrum of chemical elements present on the surface; (**d**) map of individual chemical elements.

Another activity was the analysis of the grain surface of the material accumulated on the surface of the spent moulding sand samples studied. Scanning images and maps of the elements that formed coatings on the surface of the grain matrix were taken. Figure 6 shows the results for the spent moulding sand sample labelled S1.

The analysis of the chemical composition of the elements in the observed area, in addition to the elements that form the grain matrix (quartz—SiO_2_), indicates the presence of a number of elements that may originate from the various binders used in the moulding sand. There are alkaline elements (K, Na) that may suggest an alkali-phenolic binder. The maps are elements of the type (S, Cl), which may be residues from acidic hardeners used in organic resins. The large amount of aluminium (Al) and the presence of iron (Fe), magnesium (Mg), calcium (Ca), potassium (K), sodium (Na), and titanium (Ti) may indicate the presence of a binder material such as bentonite, in whose composition they are found in varying amounts: SiO_2_ (49.9–57.6%), Al_2_O_3_ (15.9–20.2%), Fe_2_O_3_ (0.05–6.35%), FeO (0–1.0%), MgO (2.4–6.6%), CaO (0.5–3.3%), K_2_O (0–2.8%), Na_2_O (0–0.3%), and TiO (0–0.3%) [74]. There is also a significant amount of carbon (C) in the area analysed, which may originate from organic resin or as an additive of carbon dust, which is used together with the bentonite mass. In the case of the material analysed, based on the microscopic image shown in Figure 2a, it can also be seen that the grains are in varying degrees of contamination, which can indicate the mixing of, for example, different sand or moulding sand with the core mass. The sieve analysis shown in Figure 3 indicates the presence of dust in the analysed material.

**Figure 7:**
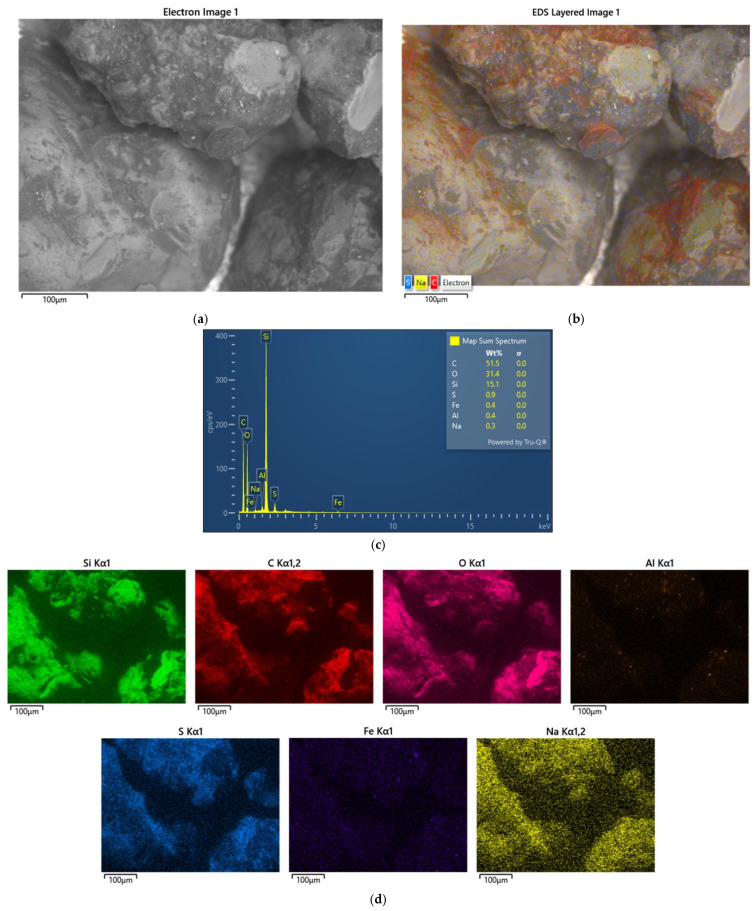
An analysis of the chemical composition of the grain surface of sample S2: (**a**) surface scanning photo, mag. ×1000; (**b**) scanning photo with elemental intensity; (**c**) spectrum of chemical elements present on the surface; (**d**) map of individual chemical elements.

Figure 7 shows a photograph taken with a scanning microscope and the results of the analysis of the grain surface of a sample of spent moulding sand marked S2.

The second of the S2 samples analysed is markedly different. Carbon (C) is the dominant component in the observed area. Sulphur (S) is also noticeable. This may indicate derivatives of an organic resin bound with an acidic hardener. The grains visible in Figure 2b have the least ‘stained’ surfaces with different intensities of discolouration, which can indicate a different degree of resin burn through depending on the distance from the mould. At the same time, as can be seen from the sieve analysis performed (Figure 4), this is spent moulding sand after pneumatic classification, as there are no finest fractions, whether matrix or crushed binder, with varying degrees of burn through.

**Figure 8:**
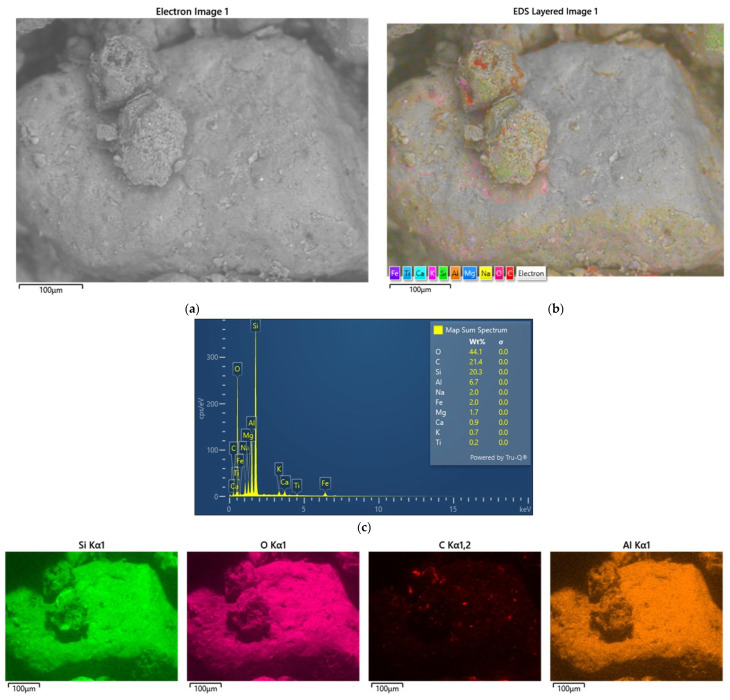

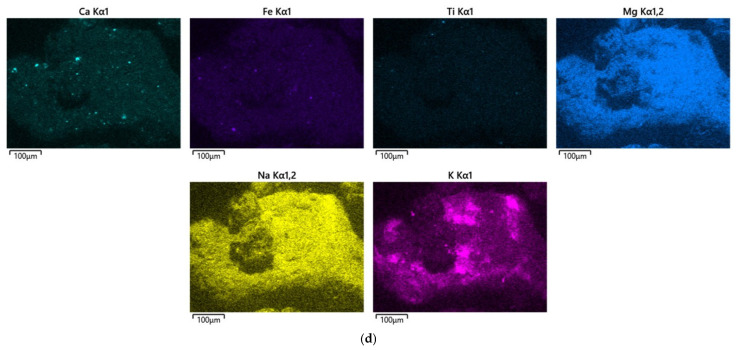
An analysis of the chemical composition of the grain surface of sample S3: (**a**) surface scanning photo, mag. ×1000; (**b**) scanning photo with elemental intensity; (**c**) spectrum of chemical elements present on the surface; (**d**) map of individual chemical elements.

Figure 8 shows the results of the last of the analysed samples of spent moulding sand S3 taken from the landfill.

In the case of the last sample tested, elements (Al, Mg, Na, K, Ca, Ti) that are constituents of bentonite, the binding material of the green sand masses, were clearly visible in quantity in the analysis of the grain surface. Carbon (C) was present in the spots. The sieve analysis shown in Figure 5 indicates that a large presence of dust, which may be derived from the coal dust present, crushed particles of the binding material, of which up to 8% is added in the case of moulding sand with bentonite [74]. This is confirmed by the observations of the digital microscope image shown in Figure 2c.

**Figure 9:**
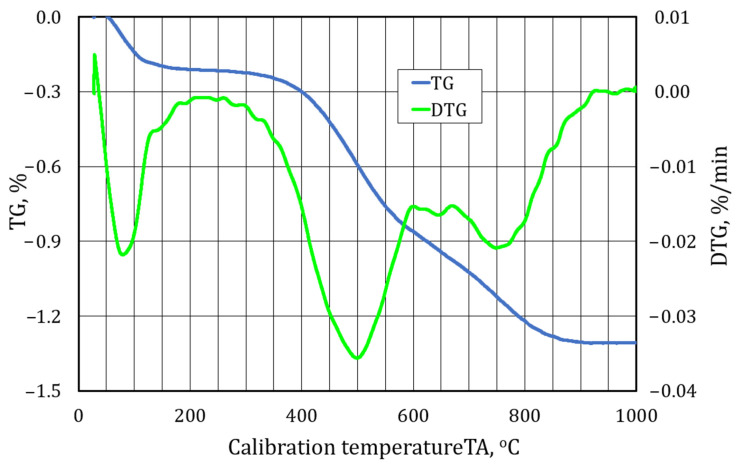
A thermogravimetric analysis of spent moulding sand S1.

**Figure 10:**
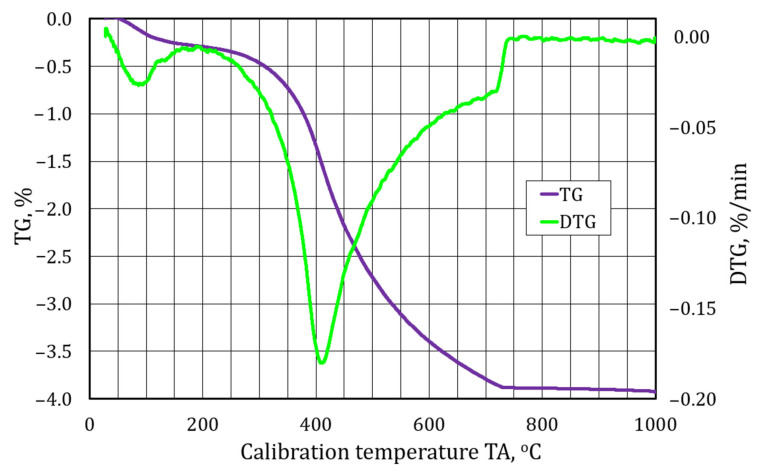
A thermogravimetric analysis of spent moulding sand S2.

**Figure 11:**
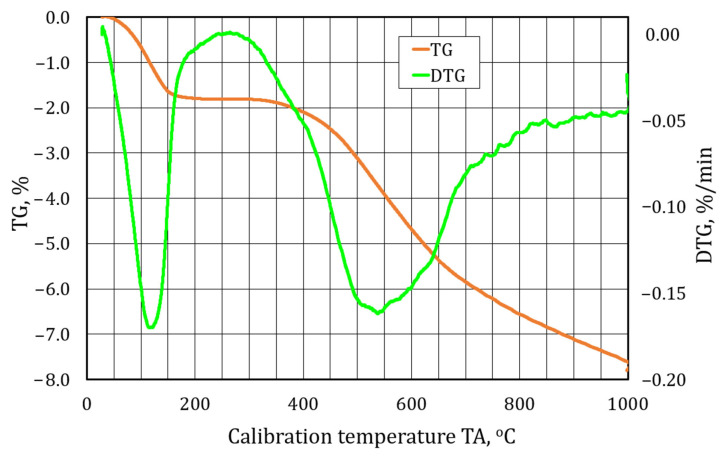
A thermogravimetric analysis of spent moulding sand S3.

A further element of this study for identifying the materials analysed was performing a thermogravimetric analysis. Figure 9, Figure 10 and Figure 11 show the waveforms obtained from the test carried out. TG is a procedure for analysing the thermal decomposition of substances, oxidation reactions, etc., associated with a change in the mass of a sample. As the change in mass on the TG curve does not fully identify the phenomena taking place, a differential method is also used, thus obtaining a differential thermogravimetric (DTG) curve. The DTG curve has its maxima and minima in the form of peaks, which characterise the temperatures at which mass changes occur at the highest rate. If the peak points downward, it indicates dehydration and thermal decomposition of the sample mass, and if it points upward, it indicates oxidation associated with the increase in the mass of the test sample.

Figure 9 shows the results of the thermogravimetric analysis of the material labelled S1.

According to the interpretation presented, the DTG curve interprets the rate at which changes in the mass of the sample of material under test occur. In Figure 9, the following peaks can be seen: The first was up to a temperature of about 170 °C, which is characteristic of the drying of the material. The second endothermic peak points downwards with a maximum at 500 °C, which may indicate the degradation of organic materials; the next small one at temperatures above 750 °C is related to the complementary final firing process, i.e., destruction. The TG curve shows that the total mass loss when heating the sample to 1000 °C is 1.32%, of which 0.2% is attributable to drying and water loss to 150 °C and 0.7% is attributable to the intensive degradation of organic material, with the remaining 0.42% associated with combustion, i.e., carbon destruction. The relatively small change in the mass of the sample indicates, in combination with the earlier analysis of the grain surface and the presence of alkaline compounds found, an alkali-phenolic binder (Figure 6). The characteristic of these binders is a noticeable kink in the TG curve above 550 °C.

Figure 10 shows a thermogravimetric analysis of the spent moulding sand S2.

In the case of the S2 material, typical curve patterns for organic binders are obtained. In the DTG curve, a first endothermic peak can be observed at 100 °C, related to the drying of the material sample. The second, however, for temperatures between 300 °C and 700 °C is related to the degradation and destruction of the organic binder. The TG curve has a characteristic pattern for organic binders; a loss of 0.2% associated with the presence of water, probably coming from moisture accumulated on the surface of the matrix grains from the way the spent moulding sand is stored in the open air; a loss of 0.5% from the degradation resin decomposition taking place; and the rest of the 3.2% mass change associated with the combustion of carbon. An additional indication that this is an organic binder is the presence of sulphur found on the surface of the matrix grains, possibly from acidic hardeners (Figure 7).

Figure 11 shows the results of the thermogravimetric analysis of the last of the S3 material samples tested.

In the case of sample S3, two characteristic peaks in the DTG curve described in the literature [74] are perceived relating to typical clay bonding materials. The first endothermic effect with an extreme at 125 °C is due to the loss of interpack water. The second in the range between 400 °C and 700 °C with a peak at 550 °C is caused by dehydration, most likely of illite, and a small one at 860 °C, possibly related to its dehydroxylation. The TG curve consists of two distinct mass losses up to 160 °C of 1.8%, and from 400 °C to 1000 °C, a mass loss of 5.7%, that is, a total mass loss of 7.5%.

**Figure 12:**
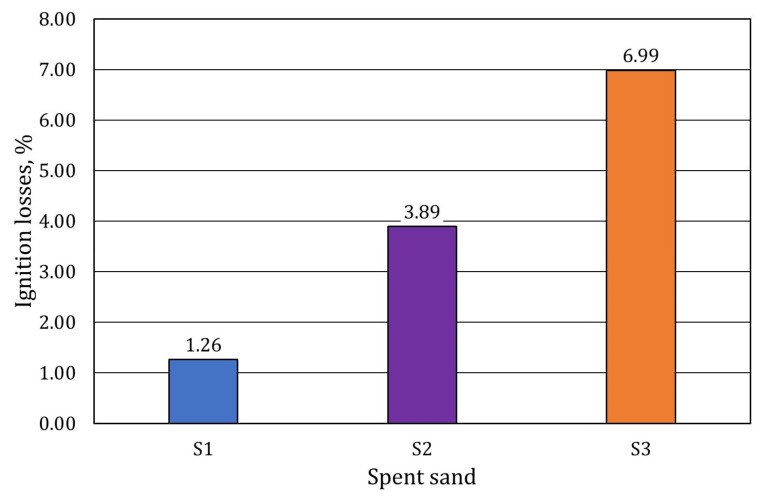
The ignition losses of the materials tested.

The next test is the ignition losses of the tested materials. The results of the test performed are shown in Figure 12.

Ignition losses indicate the varying amount of material that changes its mass as a result of roasting at 1000 °C. Most commonly, this test is dedicated to the study of masses with organic binders. And while the results obtained relate to the analysis of the grain surface of the test sample S2, where a significant amount of carbon (C) is found at 51%, there is not much of this element in the case of materials S1 and S3 (both about 21%). In the case of spent moulding sand S1, ignition losses are lower, which can be justified by the lower amount of carbon (C). In the case of S3 material, on the other hand, such high ignition losses may be related to other phenomena, such as the previously described interpack water loss or dehydration.

**Figure 13:**
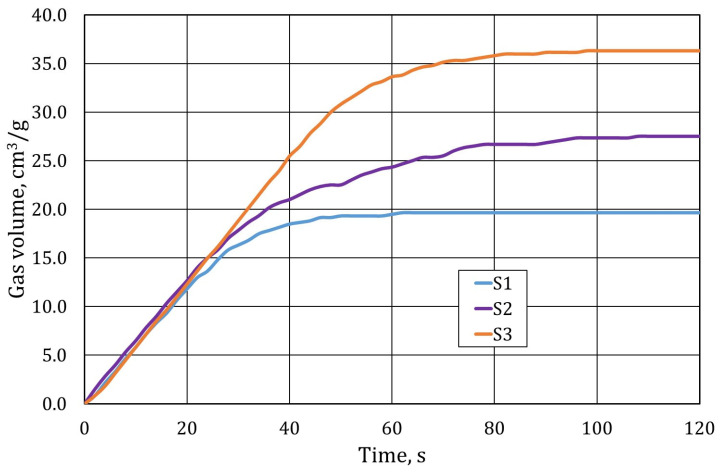
The gas-forming properties of the tested materials.

Another test carried out on samples of spent moulding sand was the gas-formability test. The results obtained are shown in Figure 13.

The gas-forming results, in terms of the amount of gas released, ranked the individual samples in the same order as the ignition losses. The lowest gas-forming capacity was found for sample S1 and the highest for sample S3, confirming other studies of carbon firing or decomposition of the binder material used at a high temperature of 1000 °C.

**Figure 14:**
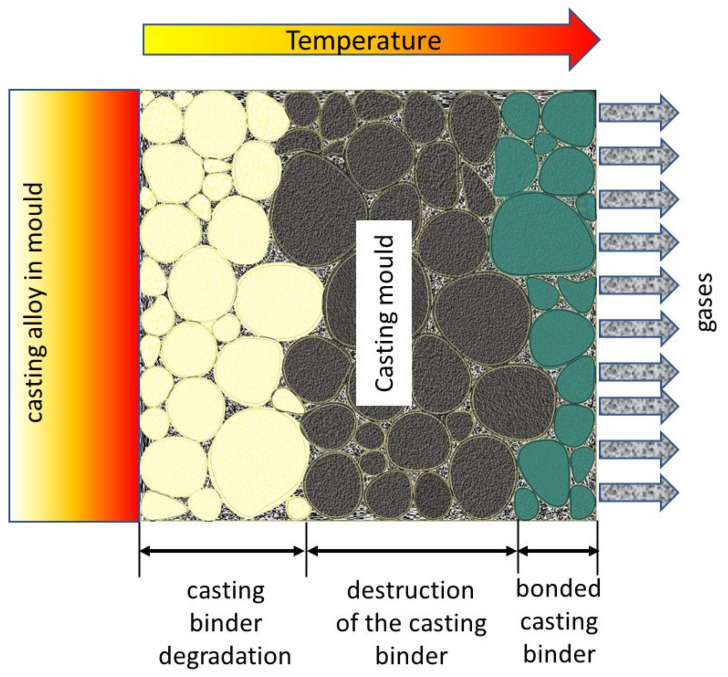
The self-regeneration effect of organic binders.

Most binders should have some thermal resistance to the liquid casting melt temperature [71], as well as the expected burning of the binder in the contact zone with the liquid casting melt, which facilitates the breaking of the casting from the mould. Sometimes, a very pronounced self-healing effect occurs. The idea of this phenomenon is illustrated in Figure 14.

### 3.2. Analysis of the Material After Thermal Treatment

The spent moulding sand in various states is regenerated after being knocked out of the mould or sent to landfill sites. The simplest technological attempt to recover the grain matrix from the spent moulding sand is thermal reclamation. This procedure allows the binder nature of the spent on the grain matrix to be verified very quickly. Therefore, samples of individual masses are subjected to thermal regeneration in an oven heated to 1000 °C for a period of 2 h, guaranteeing the effective firing of the organic compounds.

**Figure 15:**
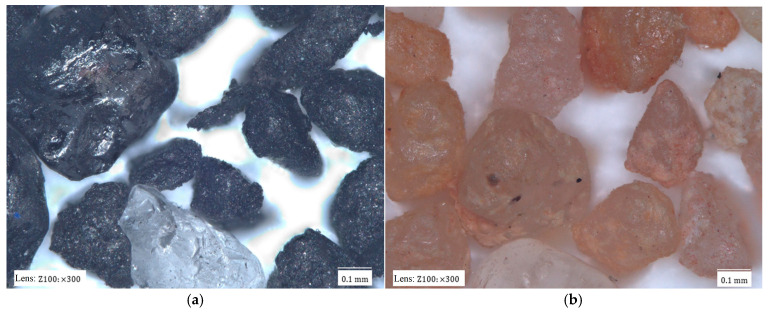
A comparison of the grain matrix of material S1, mag. × 300: (**a**) before thermal regeneration of S1; (**b**) after thermal regeneration of TS1.

Figure 15 shows a digital microscope image of the grain matrix of the sample before S1 and after the thermal regeneration of TS1.

**Figure 16:**
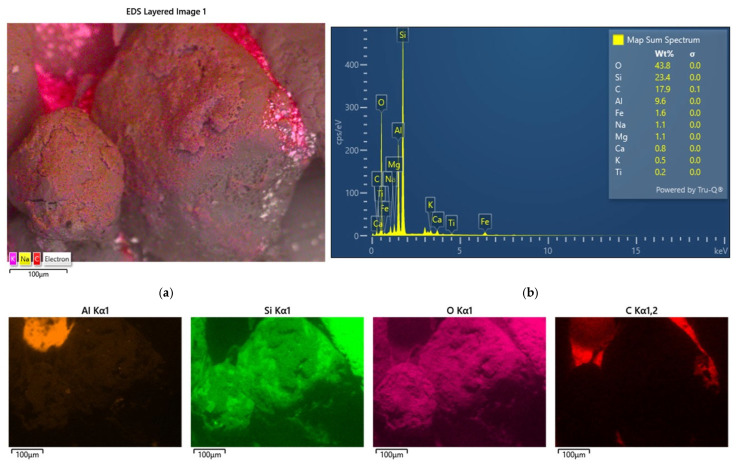

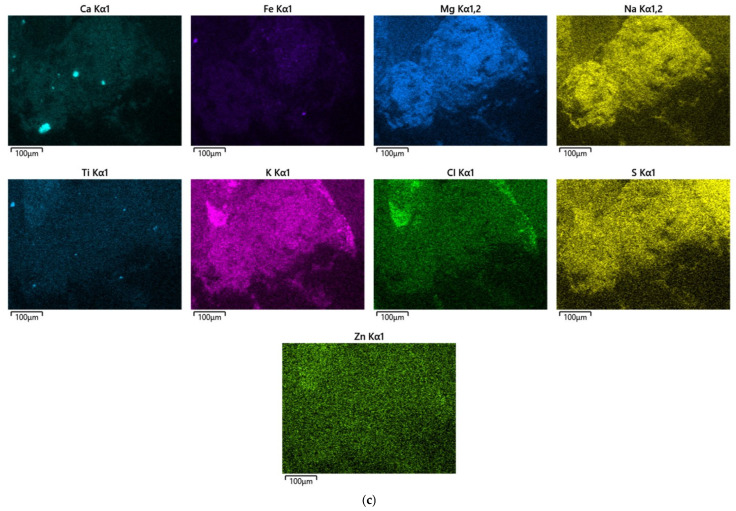
An analysis of the chemical composition of the grain surface of sample TS1: (**a**) a scanning photo with elemental intensities, (**b**) the spectrum of chemical elements present on the surface, and (**c**) maps of the occurrences of individual chemical elements.

As can be seen in Figure 15, the dark matrix grains of the S1 material, after the thermal regeneration of TS1, change colour to a light colour. However, the resulting surface of the TS1 matrix grains is not clean. Inorganic residues can be seen on the surface, which have not been burnt off. Therefore, an analysis of the grain surface of the matrix coating material is performed using a scanning microscope. The results obtained are shown in Figure 16.

**Figure 17:**
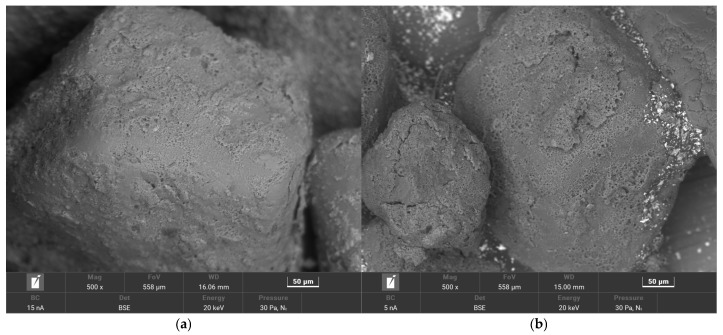

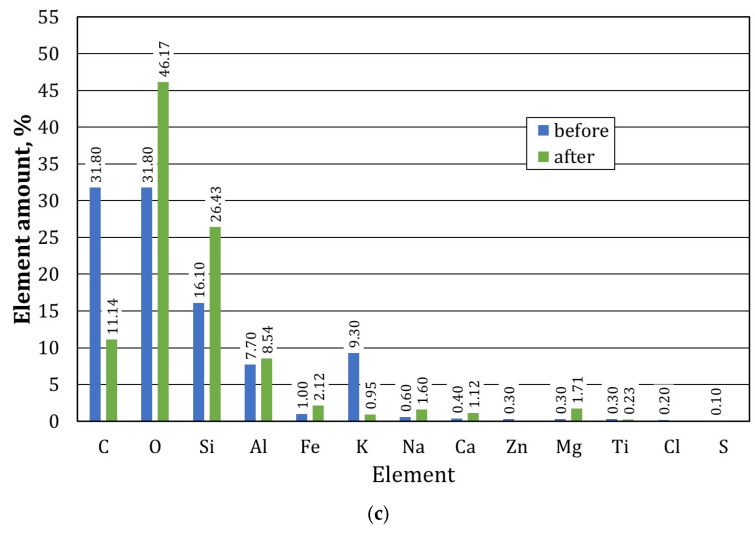
A comparison of the grain matrix before S1 and after thermal regeneration treatment of the TS1: (**a**) image of grain matrix S1, scanning microscope, mag. ×500; (**b**) image of the grain matrix TS1, scanning microscope, mag. ×500; (**c**) comparison of chemical composition in grain matrix S1 and TS1.

After thermal regeneration treatment, a significant amount of inorganic elements was observed in the TS1 grain matrix sample. To determine the extent of the changes with respect to the starting material S1, a summary was made and is shown in Figure 17. Additionally, the S1 and TS1 matrix grains were imaged using a scanning microscope.

**Figure 18:**
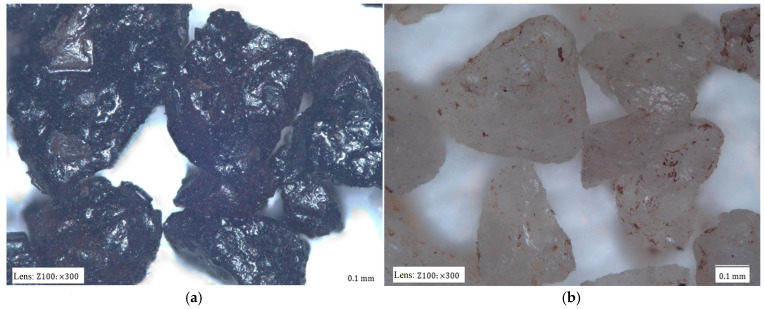
A comparison of the grain matrix of material S2, mag. ×300: (**a**) before thermal regeneration of S2; (**b**) after thermal regeneration of TS2.

The thermal regeneration process did not remove the material that accumulated on the grain surface. Figure 17b shows the remains of the spent binder, which is no longer fused to the grain matrix (see Figure 17a). This state of affairs resulted from the burning of the carbon (C) present in the S1 material sample and the gasification of possibly some of the compounds present in the composition of the spent binder. The burning of the organic material resulted in an increase in other elements in the analysis of the grain surface, both the grain matrix itself (Si, O) and other inorganic elements (Al, Fe, Na, Ca, Mg). In the case of this material, combined thermal–mechanical regeneration could be used to recover the grain matrix, during which the organic compounds could be burnt off first and then the combustion products and the remaining inorganic material could be mechanically removed. Figure 18 shows digital microscope images of the initial S2 grain matrix and, after thermal regeneration, of the TS2 grain matrix.

The image of the grain matrix before S1 and after the thermal regeneration of TS2, shown in the figure, indicates the very good cleaning of the spent moulding sand from the binder as a result of the thermal regeneration procedure carried out. A clean grain surface and few residual combustion products can be clearly seen.

**Figure 19:**
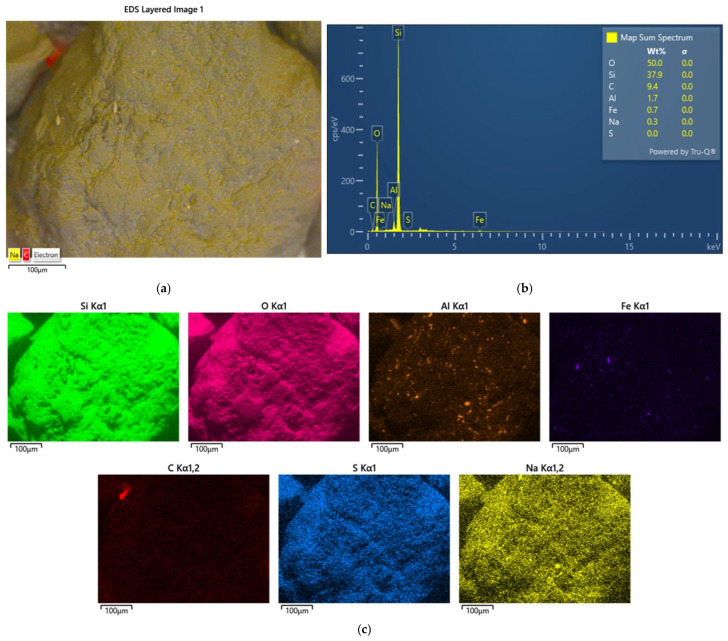
An analysis of the chemical composition of the grain surface of sample TS2: (**a**) a scanning photo with elemental intensities, (**b**) the spectrum of chemical elements present on the surface, and (**c**) maps of the occurrence of individual chemical elements.

An analysis of the grain surface was performed for this material, and the results obtained are shown in Figure 19.

**Figure 20:**
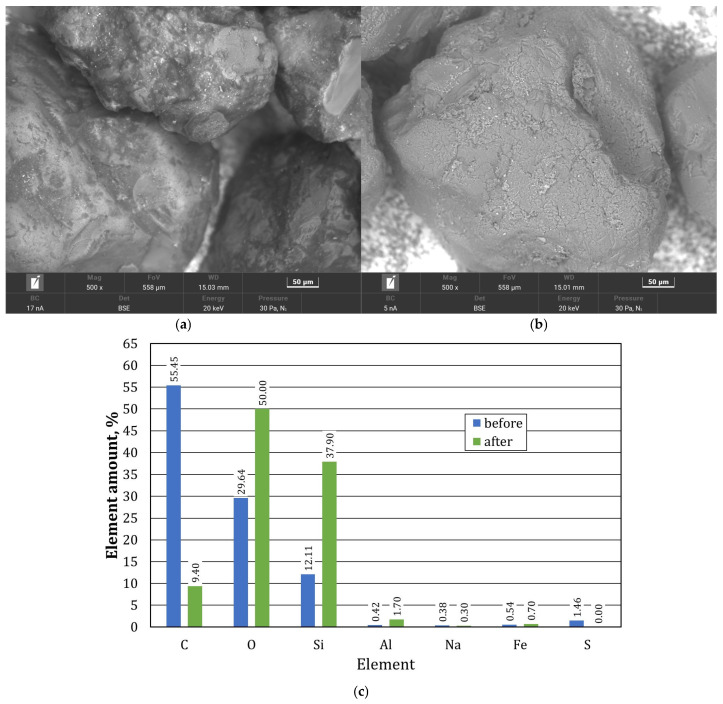
A comparison of the grain matrix before S2 and after thermal regeneration treatment of the TS2: (**a**) image of grain matrix S2, scanning microscope, mag. ×500; (**b**) image of the grain matrix TS2, scanning microscope, mag. ×500; (**c**) comparison of chemical composition in the grain matrixes S2 and TS2.

After thermal regeneration treatment, an increase in the number of grain matrix-forming elements was observed on the TS2 grain matrix sample (Figure 19b). Scanning images of the grain matrix samples were compared. To quantify the quantitative change in the resulting TS2 material compared to the initial S2 material, a comparison was made, as shown in Figure 20.

The thermal regeneration process mostly removed the spent binder from the grain surface. However, small amounts of it remained in irregularities on the surface of the quartz sand. Figure 20b shows the presence of additional material in the depressions. In addition, it is possible to see in some places the rather worn surface of the quartz matrix itself, which can be compared to an orange peel. In other places, the matrix grain has a smooth crystalline surface. Figure 20c compares the chemical compositions determined on the grain matrix surfaces of the S2 and TS2 matrixes. Thermal regeneration significantly reduced the amount of carbon (C) in the test material. This indicates that the binder that coated the grains of the test material was organic, so a dedicated method of recovering the quartz matrix may be thermal regeneration.

**Figure 21:**
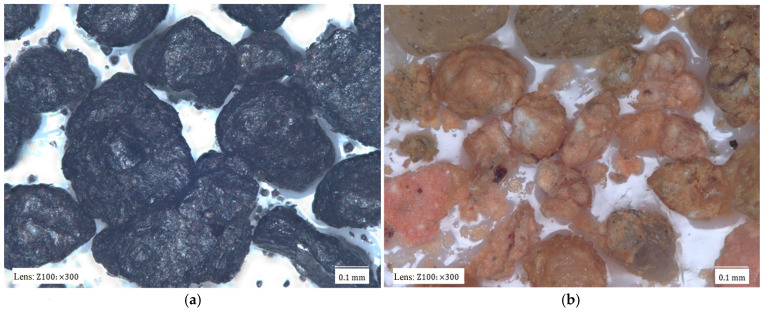
A comparison of the grain matrix of material S3, mag. ×300: (**a**) before thermal regeneration of S3; (**b**) after thermal regeneration of TS3.

An analogous comparison was made, as for the samples discussed samples, for the initial grain matrix S3 and, after thermal regeneration, TS3. Figure 21 shows images taken with a digital microscope of both samples.

**Figure 22:**
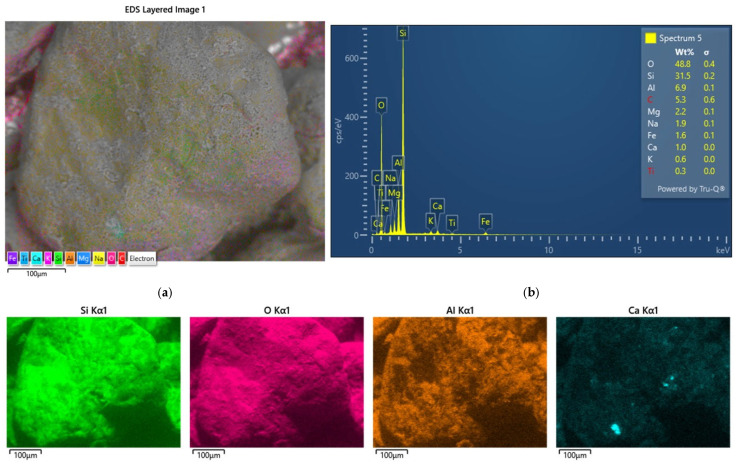

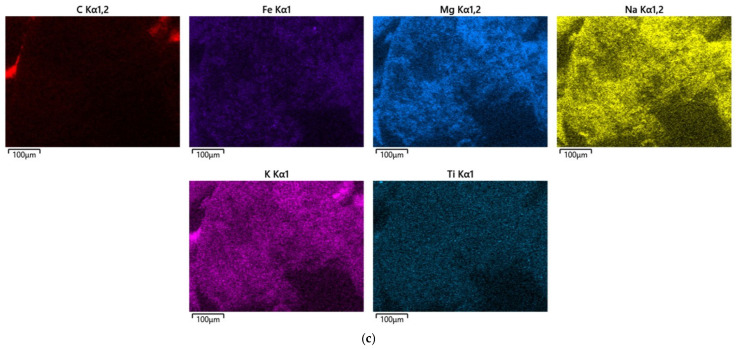
An analysis of the chemical composition of the grain surface of sample TS3: (**a**) a scanning photo with elemental intensities, (**b**) the spectrum of chemical elements present on the surface, and (**c**) maps of the occurrence of individual chemical elements.

In the case of this material, thermal regeneration did not yield satisfactory results. The matrix grains visible in Figure 21b are covered by a layer of sintered spent bonding material. An analysis of the chemical composition of a sample of TS3 material was performed, as for the previous materials, and the results are shown in Figure 22.

Figure 22 shows the spectrum and distribution of chemical elements in the form of intensity maps. The distribution maps show the elements magnesium (Mg), sodium (Na), and potassium (K) with particular intensity.

**Figure 23:**
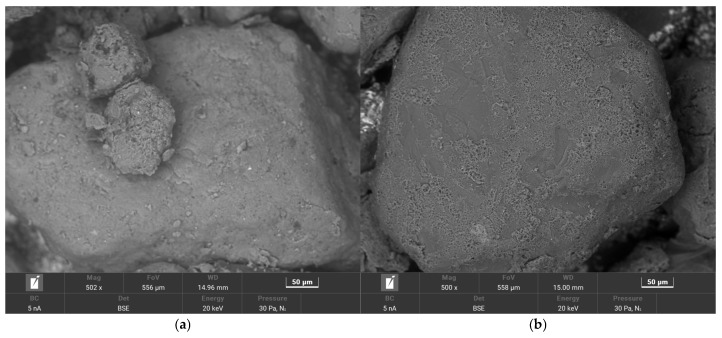

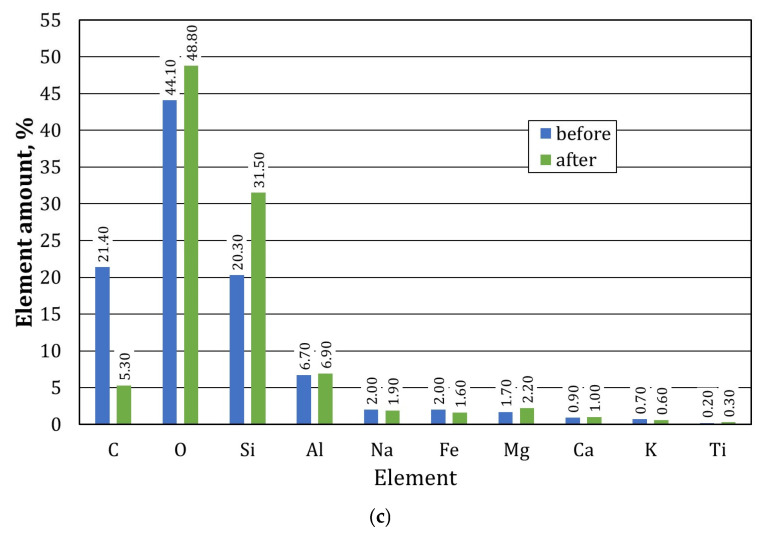
A comparison of the grain matrix before S3 and after thermal regeneration treatment of the TS3: (**a**) an image of grain matrix S3, scanning microscope, mag. ×500; (**b**) an image of the grain matrix TS3, scanning microscope, mag. ×500; (**c**) a comparison of chemical composition in grain matrixes S3 and TS3.

Figure 23 shows a comparison of how much thermal treatment affected the chemical composition of the elements accumulated on the grain surface for the S3 starting material.

In Figure 23b, it can be observed that the form of the accumulated bonding material has changed as a result of the high temperature compared with the starting material (Figure 23a). The material appears porous. This form could be the result of the gasification or burning of parts of the binder. Figure 23c compares the chemical compositions of the elements accumulated on the surfaces of the S3 and TS3 grain matrixes after thermal regeneration. The results show a significant loss of carbon (C), an increase in the contents of grain matrix elements (Si, O), and comparable amounts of the other elements analysed. The action of temperature, for example, on the clay causes its deactivation and oolitisation, transforming the binding material into chamotte. The chemical composition of the chamotte in terms of the presence of individual elements is consistent with that shown in Figure 23c. In the case of this material, combined thermal–mechanical regeneration can also be effective in cleaning the grain matrix of the binding material.

## 4. Discussion

The results presented here were intended to provide an answer as to whether spent moulding sand of unknown origin (unknown grain matrix, unknown binding agent used) could be a source of grain matrix for reuse. Using thermal treatment as a first step, it is easiest to eliminate the residual by burning any organic substances that could be present in the unknown material. Over the course of several decades, with the development of organic chemistry, various organic binders emerged and were commonly introduced into foundry technology. Their popularity in many cases was due to the speed and ease with which they can be used to cast moulds, which contributed to increased production efficiency. Organic binders in casting technology improved important parameters, such as the knock-out property of the moulding sand from the box and the separation of the casting from the moulding sand. These parameters were very good in the case of binders that burnt out in contact with the liquid metal. This is graphically illustrated in Figure 14. Therefore, organic binders replaced, in many cases, binders of natural origin (e.g., bentonites), which did not guarantee such parameters. This was the first reason many spent moulding sand samples with inorganic binders ended up in landfills. Another element influencing the transfer of spent moulding sand to landfill was the change from the organic binder already in use to another. For general ecological reasons and the high arduousness of organic binders used at workplaces, furfuryl resin (the so-called furan sand) was replaced with less arduous binders (e.g., alkali-phenolic). The replacement of all of the moulding sand samples used by a given foundry was associated with a change from a furan sand binder with an acidic pH to an alkaline-phenolic binder with an alkaline pH. In the foundry industry, it should be emphasised that it is a common rule that, as a result of one of the methods of regenerating spent moulding sand, the recovered grain matrix is used in the same technology. The rationale presented for the change in technology has resulted in significant amounts of various spent moulding sands being deposited in landfills, which are currently already difficult to identify, as their origin is often unknown.

In most cases, reclamation procedures are carried out on known spent moulding sand used in individual foundries. The research undertaken in this study is a novelty in the field of managing a significant amount of grain matrix already in landfills. The research carried out is an attempt to identify the materials acquired therein and to find a suitably effective method to recover the pure quartz matrix. This is being conducted in accordance with the expectations of companies that would like to use such material. Hence, the following suggestions are made regarding the possibility of taking a specific action on the materials investigated in order to reuse them:

Spent moulding sand, S1, is probably a mixture of moulding and core sand (matrix grains covered with a binder to various degrees). For this material, the smallest technologically unsuitable fractions should be removed first. In the next stage, a combined thermal–mechanical reclamation will be necessary. First, we would use thermal reclamation to remove organic compounds. Then, we would mechanically remove inorganic compounds. The process should be complemented by pneumatic classification. As a result of the irregular shape of the matrix grains, the acquisition of high purity quartz sand may prove to be a time-consuming and costly process with limited yields. However, it appears that this material may be useful for further applications, whether in a foundry or other industries where pure quartz is not needed.

The spent moulding sand S2, the tests showed, is likely to be with an organic binder bound by an acidic hardener (the presence of sulphur). After thermal regeneration, small amounts of impurities are visible on the surfaces of the grains. This condition is due to the binder firing under static conditions (immobile bed). In the case of this material, thermal regeneration in a fluidised bed may be sufficient to remove the spent binder. During this process, the matrix grains, due to their different sizes, also interact by rubbing against each other during mixing. In addition, since the process is carried out in a fluidised bed, in which the grains are additionally cleaned of the products of combustion or mutual abrasion, a high-purity grain matrix should be obtained. A grain matrix thus cleaned could be reused in the foundry.

The S3 contaminated mass appears to be the most difficult material to clean. Assuming that the binder in the test material is clay, it is best to use a wet reclamation procedure. However, such a mass may already have a zooliticised part of the binder material, which requires abrasive processes. Therefore, a combined three-stage reclamation would be appropriate in this case. First, mechanical reclamation (initial removal of bentonite and coal dust, pneumatic classification), then thermal reclamation (combustion of organic compounds), and finally mechanical reclamation again and pneumatic classification (removal of the chamotte layer, the zoolitised layer, resulting from bentonite deactivation). For this material, the inputs are the largest and the area of application is in a foundry or in construction.

## 5. Conclusions

The following conclusions can be drawn from studies carried out on spent moulding sand taken from landfills:

The recovery of pure quartz matrix from spent moulding sand is a very difficult task. Depending on the type of binder material used, different regeneration methods are used. If the binders are inorganic, the recovery of the grain matrix can be achieved by mechanical methods. However, because of the varied shape of quartz sand and its undulating surface, it is almost impossible to clean 100% of the impurities mechanically. As a rule, impurities accumulate in the depressions of the surface, hence the great difficulty in removing them. Mechanical treatment would have to grind the grain matrix into a ball to access its entire surface. This would require a large energy input and would therefore involve high process costs, with a high loss of machined material.

In the case of organic binders, the recovery of the pure grain matrix is easier. If the correct regeneration temperature is ensured, the organic material can be effectively burnt off and any resulting combustion products removed by abrasion during the fluidised bed regeneration process.

A more difficult challenge arises when the binder is mixed in nature, e.g., alkali-phenolic; i.e., we have both inorganic and organic material in the binder. Thermal regeneration will remove the organic compounds, while on the surface of the grains, often in the aforementioned surface depressions, inorganic elements accumulate, which are very difficult to remove. The situation is similar when we have moulding sand with an inorganic binder mixed with core sand with an organic binder.

Therefore, it is common in the foundry industry to use the regenerate of a given technology in the same process. The mixing of regenerates is not recommended as a result of the varying chemical natures of the different binders used.

At the present stage of technology, the recovery of pure quartz sand from spent moulding sand for other applications appears to be unattainable in most cases. However, given that newer material processing (recycling) technologies are emerging, spent moulding sand from landfills may prove to be a valuable material for various applications in the future, given the depletion of its natural sources.

## Data Availability

The original contributions presented in this study are included in the article. Further inquiries can be directed to the corresponding author.
